# Thomas H. Chandler, A.B., D.M.D.

**Published:** 1895-11

**Authors:** 


### Obituary.

---

### THOMAS H. CHANDLER, A.B., D.M.D.

By the death of Thomas H. Chandler, A.B., D.M.D., the American Academy of Dental Science has lost a Fellow of rare worth, one who had the respect of the profession at home and abroad, and the love of all who knew him intimately.

He became a Fellow of this Society in 1870, and was its president in 1881. Having taken a collegiate course before studying the profession of dentistry, he was well calculated to take the high stand which he has always maintained in regard to dental education. Believing, as he did, that general culture was a better foundation upon which to build a dental education than was the technical training of a medical course, he is to be honored for his efforts to make ours a liberal profession. He was ever ready to assist those who sought his aid, and took pleasure in acquiring knowledge that he might, in turn, impart it to others.

His genial and kindly disposition made his association with us peculiarly pleasant. His high ideal of what the dentist should be is an example worthy of our emulation. His dignified and courtly bearing, his scholarship, and his devotion to his work did much to elevate dentistry to its present honorable position. His aim has been high; his achievements great; his record honorable; and dentistry will forever bear the impress of his life.

Be it therefore

*Resolved*, That in the death of Professor Chandler the Fellows of this Academy feel that they have sustained a great and personal loss, and the dental profession that of one of its most eminent members.

That to his family we extend most sincere and heart-felt sympathy.

That a page of our records be set aside in honor and affection to his memory.

C. P. Wilson,
Dwight M. Clapp,
R. R. Andrews.

Boston, October 2, 1895.

